# Rare variants in *NEUROD1* and *PDX1* are low penetrance causes of MODY, whereas those in *APPL1* and *WFS1* are not associated with MODY

**DOI:** 10.2337/db25-0442

**Published:** 2025-08-08

**Authors:** Aparajita Sriram, Matthew N. Wakeling, Andrew T. Hattersley, Michael N. Weedon, Kevin Colclough, Thomas W. Laver, Kashyap A. Patel

**Affiliations:** 1 Department of Clinical and Biomedical Science, University of Exeter, Exeter, UK; 2 Royal Devon and Exeter NHS Foundation Trust

## Abstract

An accurate genetic diagnosis of Maturity-Onset Diabetes of the Young (MODY) is critical for personalised treatment. To avoid misdiagnosis, only genes with strong evidence of causality must be tested. Heterozygous variants in *NEUROD1, PDX1, APPL1*, and *WFS1* have been implicated in MODY, but strong genetic evidence supporting causality is lacking. We therefore assessed their existing genetic evidence and performed the gene-level burden tests in a large MODY cohort, alongside two established MODY genes as positive controls (*HNF1A*-high penetrance, *RFX6* -low penetrance).

The first reported MODY-associated variants in *NEUROD1, PDX1, APPL1*, and *WFS1* were <1:20,000 frequency. Based on the small number of large published pedigrees per gene (n<3), MODY-associated variants showed only modest co-segregation in these genes. Crucially, ultra-rare (MAF<1:10,000) protein-truncating and predicted-damaging missense variants in *APPL1* and *WFS1* were not enriched in a MODY cohort (n=2,571) compared to population controls (n=155,501; all p>0.05). In contrast, variants in *NEUROD1* and *PDX1* were enriched, albeit at levels comparable to *RFX6*. Multiple sensitivity analyses corroborated these findings. In summary, rare heterozygous variants in *NEUROD1* and *PDX1* are low-penetrance causes of MODY, while those in *APPL1* and *WFS1* lack robust genetic evidence for causality and should not be included in MODY testing panels.

## Introduction

An accurate genetic diagnosis of Maturity Onset Diabetes of the Young (MODY) is vital for patients but requires knowledge of the causal genes to prevent misdiagnosis. MODY is the most common form of monogenic diabetes, responsible for ~3% of all diabetes diagnosed under 30 years old (1). MODY is an autosomal dominant, genetically heterogeneous disease with 16 genes implicated to date (2). A genetic diagnosis optimises treatment, for example, individuals with HNF1A-MODY respond well to sulfonylurea tablets (3). Currently, genetic testing is primarily conducted using a multi-gene panel approach, where all relevant genes are analysed in a single assay. However, testing genes with limited evidence of causality increases the risk of misdiagnosis and inappropriate clinical management.

Re-evaluating genetic evidence can identify MODY genes without sufficient evidence for causality. We recently demonstrated that variants in *BLK, KLF11*, and *PAX4* reported to cause MODY are common in the population, have limited co-segregation, and lack enrichment in MODY cases compared to controls (4). Our approach leveraged large population databases such as the UK Biobank (5) and gnomAD (6) which were not available when these genes were published. This evidence contributed to the ClinGen international consensus panel (7) reclassifying the gene-disease relationships as refuted.

*NEUROD1, PDX1, APPL1* and *WFS1* have limited evidence of causality and need re-evaluation. Recently ClinGen identified *NEUROD1, PDX1* and *APPL1* as MODY genes where further genetic evidence is needed to either support or refute their pathogenicity (8). These genes were either published before large-scale population data was available and/or lacked ultra-rare variant enrichment analysis in MODY cases against population controls. This is also the case for heterozygous variants in *WFS1. WFS1* is a well-established cause of monogenic diabetes. However, the causal variants are either recessive loss-of-function causing Wolfram syndrome or specific *de novo* missense variants causing a syndrome of neonatal diabetes, deafness and cataracts (9). Dominant variants have been reported to cause adult-onset diabetes (10) but lack robust genetic evidence to support pathogenicity. Re-evaluating the evidence of causality in these genes is important to prevent misdiagnosis.

In this study, we used a large clinically suspected MODY cohort and population controls to assess the genetic evidence for *NEUROD1, PDX1, APPL1*, and *WFS1*.

## Research Design and Methods

### Study populations

#### MODY cohort

This cohort comprises 2,571 unrelated individuals of European ancestry with clinically suspected MODY from routine clinical practice, referred for genetic testing to the Exeter Genomics Laboratory (clinical features in Supplementary Table 1). The North Wales Ethics committee approved the study (no. 17/WA/0327). We obtained informed consent from probands or guardians.

#### UK Biobank

The UK Biobank is a population-based research initiative in the UK that recruited participants between the ages of 40 and 70. It has extensive phenotypic data along with comprehensive genetic information. For this study, we included individuals with genome sequencing data from the 200,000 genomes release of the UK Biobank. All participants provided informed consent, and the UK Biobank resource was approved by the UK Biobank Research Ethics Committee.

#### GnomAD

We used the Genome Aggregation Database (gnomAD) version 3.1.2 (6), which includes genome sequencing data from 76,156 individuals, as an alternative population control for a sensitivity analysis. GnomAD aggregates genetic data from various sequencing projects and makes it available to the wider scientific community.

### Genetic testing

#### MODY cohort

The probands underwent targeted next-generation sequencing for a panel of monogenic diabetes genes using a previously described method (11). We annotated variants using Alamut Batch 1.11 (Interactive Biosoftware, Rouen, France) against Genome Reference Consortium Human Build 37 (GRCh37) and lifted them over to Genome Reference Consortium Human Build 38 (GRCh38), to compare them with our controls. We examined the following RefSeq transcripts for our genes of interest: *APPL1* NM_012096.3, *HNF1A* NM_000545.8, *NEUROD1* NM_002500.5, *PDX1* NM_000209.4, *RFX6* NM_173560.4, and *WFS1* NM_006005.3.

#### UK Biobank

We included whole genome sequencing data from 155,501 unrelated individuals of European ancestry in the analysis. We focused on genome sequencing over exome sequencing data due to its well-described advantage of uniform coverage of coding exons to reduce the technical artefacts in our analysis (12) The sequencing methodology for UK Biobank is described in detail by Szustakowski *et al*. (13) and is available from https://biobank.ctsu.ox.ac.uk/showcase/label.cgi?id=170. Variant-calling was performed against genome build GRCh38. Variants were annotated using Alamut Batch 1.11 (Interactive Biosoftware, Rouen, France).

#### GnomAD

We included whole genome sequencing data from 34,029 Non-Finnish Europeans from gnomAD v3.1.2 to match with our MODY cohort. The gnomAD consortium conducted joint variant calling across samples using a standardized BWA-Picard-GATK pipeline. GnomAD performed quality control and analysis with the Hail open-source framework for scalable genetic analysis (6). Variants from gnomAD v3 were called against GRCh38. We annotated the gnomAD variants using Alamut Batch 1.11 (Interactive Biosoftware, Rouen, France).

#### Ancestry

We determined ancestry in the MODY cohort using an implementation of the LASER method for targeted panel data (14).

### Quality Control

#### MODY cohort

We implemented a rigorous quality control (QC) process at the sample, variant, and genotype levels to ensure the integrity of sequencing data obtained from different sequencing technologies. Given the potential discrepancies that arise when comparing sequencing data from multiple sources, applying stringent QC measures was essential to minimize errors and enhance reliability. We removed six samples due to higher missingness of >2%. We performed multiple QC steps at variant level that included removing variants with (A) an FS (Phred-scaled p-value using Fisher’s exact test for strand bias) greater than 60, (B) a QD (Variant Confidence/Quality by Depth) lower than 2, (C) a ReadPosRankSum (Z-score from the Wilcoxon rank sum test of Alt vs. Ref read position bias) lower than -8, and (D) an MQRanksum (Z-score from the Wilcoxon rank sum test of Alt vs. Ref read mapping qualities) lower than -12.5. We also removed variants with a (E) mapping quality lower than 40, a (F) read depth below 20, or (G) a genotype quality below 20. Additionally, we filtered out variants with allelic imbalance, if a binomial test of detecting allelic imbalance yielded a p-value less than 0.001. We excluded variants in the *HNF1A* c-insertion region (12:120994310-120994335) due to high false-positive rates. Finally, we removed the genotypes with a missingness rate exceeding 2%.

After implementing all QC measures, we retained 660 high-quality variants, ensuring a robust dataset for downstream analysis.

#### UK Biobank

We performed the same QC in the UK Biobank with minor changes to reflect it had genome sequencing rather targeted gene panel data. The changes included lowering the read depth to 15 for excluding the variants. We also removed variants which had poor AA score (< 0.5). The AAscore is a quality metric that estimates the probability of a variant being a true positive, generated by Graphtyper (15)Finally, we removed variants which are flagged as being located in low-complexity regions by gnomAD to remove potentially false positive variants. Any variant that failed QC in either MODY or UK biobank were removed from both cohorts for the analysis.

#### GnomAD

Gnomad has already performed sample and variant QC. In addition to this, we excluded variants if they were located in regions with low coverage (≤10× in ≤80% of samples). We also excluded variants that were filtered by gnomAD and also removed variants if they were flagged as being located in a low complexity region in gnomAD. Any variant that failed QC in either the MODY cohort or gnomAD were removed from both cohorts for the analysis.

### Ultra-rare variant burden analysis

We performed ultra-rare variant (MAF <1:10,000) gene-level burden tests between the MODY cohort, UK Biobank controls and gnomAD v3.1.2 controls. We analyzed protein-truncating variants (PTVs), damaging missense variants defined using a REVEL score cutoff >0.7 (16) and synonymous variants in each gene. PTVs included stop-gain, splice site and frameshift variants. The primary analysis excluded PTVs expected to avoid nonsense mediated decay (NMD): those in the last exon and the last 50 base pairs of the penultimate exon. All PTVs in *NEUROD1* were included in the analysis because this is a single exon gene. We used the fisher’s exact test to assess the association. We also computed odds ratios (ORs) with 95% confidence intervals (CIs) to quantify effect sizes.

We used synonymous variants to assess test inflation and potential technical artefacts across cohorts. As positive controls in our analysis, we included *HNF1A* variants, representing a high-penetrance MODY gene, and *RFX6* variants, representing a low-penetrance MODY gene. To account for multiple testing, we applied a Bonferroni-corrected p-value threshold of 0.002 (0.05 / (6 genes × 3 groups)).

We performed power calculations to assess the detectable odds ratios at different minor allele frequencies. For variants with a MAF of 1 in 10,000, we had greater than 80% power to detect odds ratios as low as 9.8. At a MAF of 1 in 20,000, we maintained over 80% power to detect odds ratios of 14.5 or greater. For more common variants with a MAF of 1 in 5,000, we had sufficient power to detect odds ratios as low as 6.7.

### Sensitivity Analysis for Burden Testing

We performed multiple sensitivity analyses. This included burden testing at both a stricter allele frequency threshold (MAF<1:20,000) and a more lenient threshold (MAF<1:5,000) to ensure that our findings were not dependent on the exact frequency cutoff chosen. Additionally, we conducted burden tests using gnomAD v3.1.2 controls as a sensitivity analysis, to test whether our results remained consistent with an independent control dataset. To further investigate potential region-specific effects, we performed burden tests focusing on functional domains within genes (Supplementary Methods). Finally, we conducted burden tests restricted to NMD escape regions to explore an alternative mechanism of disease.

### Re-evaluation of published data

#### Variant frequencies

We assessed the frequencies of the first published variants in our genes of interest. To check variant frequencies, we used gnomAD v2.1.1, which includes sequencing data from 141,456 individuals but excludes UK Biobank data to prevent overlap with our burden test analyses. The most common pathogenic MODY variant with at least 50% penetrance should appear no more than seven times in gnomAD v2.1.1 (frequency < 4.9 × 10^−5^) as per the framework from Whiffin *et al*. (17), using the MODY prevalence of 248 cases per million (18). However, it is important to note that variants in less common genes, like those analyzed in this study, which only contribute to a small proportion of MODY and have high variant heterogeneity, are expected to be much less frequent (19).

#### Co-segregation analysis

We reviewed all published pedigrees for putative MODY variants in our genes of interest to evaluate co-segregation, which measures how often a variant and disease are inherited together within a family. We followed ClinGen guidelines for this analysis and included only large pedigrees with four or more co-segregations (20). To avoid confounding the co-segregation results with common variants, we restricted our analysis to variants with a frequency below 1 in 1,500 in gnomAD v2.1.1. This exclusion criterion removed four pedigrees (21–24). We performed binomial tests to assess whether co-segregation (the proportion of family members with diabetes and the variant) and penetrance (the proportion of family members with the variant but without diabetes) were significantly different from the expected values of 50% and arbitrary threshold of 10%, respectively. The proportion of family members with diabetes and variant was calculated as the number of family members with both diabetes and variant divided by the total number of family members with diabetes and genotype information. The proportion of family members with the variant but without diabetes was calculated as the number of family members without diabetes with variant divided by the total number of family members without diabetes and genotype information who are at least 25 years of age.

## Data and Resource Availability

UK Biobank data is available at https://www.ukbiobank.ac.uk/enable-your-research and is accessible through application. GnomAD data is freely available to all at https://gnomad.broadinstitute.org/. The MODY cohort data is not publicly available for ethical and patient confidentiality related reasons but is available upon reasonable request to the corresponding author. No applicable resources were analyzed or generated during the study.

## Results

### The first reported variants in *NEUROD1, PDX1, APPL1* and *WFS1* are rare in the population

Variants reported to cause MODY should be rarer than the disease itself. Therefore, we assessed the frequencies of the first published MODY variants in 141,456 individuals from gnomAD v2.1.1. We used *HNF1A* and *RFX6* as positive controls, representing highly penetrant and lower penetrant MODY genes, respectively. The first-reported MODY-causing variants in our high penetrance control *HNF1A* are all rare (allele frequency < 1x10^-5^ in gnomAD v2.1.1) (25). This is also the case for our low penetrance control *RFX6*, except for the Finnish variant, p.H293Lfs, which is relatively common in the Finnish population (allele frequency of 2x10^-3^ in the Finnish population in gnomAD v2.1.1) (Table 1) (26) The first published variants in *NEUROD1* (27), *PDX1* (28), *APPL1 (29)* and *WFS1* (10) are all rare in the population (allele frequency < 3x10^-5^) (Table 1). However, the first *APPL1* variants were published after the release of the first large population genetic database, ExAC (30,31), so would only have analyzed rare variants (29). The first *WFS1* variant shown to cause MODY was reported in a Finnish family before gnomAD but remains absent in 12,562 Finnish individuals in gnomAD (10). The *PDX1* variant was only seen in two individuals, and the *NEUROD1* variant was present in six individuals but filtered out in GnomAD2.1.1. (27,28).

### All four genes show co-segregation in published pedigrees

Highly penetrant MODY-causing variants are expected to co-segregate with diabetes within families. Following ClinGen guidelines (20), we analyzed large published pedigrees with at least four co-segregations of the variant with the disease. We did not identify any additional published pedigrees for *APPL1, WFS1* and *PDX1* apart from the original publications. The combined logarithm of the odds (LOD) score for three families with three different *NEUROD1* variants (27,32,33) was 8.1 (Table 2). For *PDX1*, a single family showed a LOD score of 3.4 (28). *APPL1* pedigrees had a LOD score of 3.3 (26). *WFS1* demonstrated co-segregation, though with a lower LOD score of 2.1 based on one pedigree (10) (Table 2). In all these pedigrees, more than 50% of family members with diabetes had a variant (all binomial p < 0.04). However, despite its LOD score being comparable to the low-penetrance control, in the *APPL1* pedigree, more than 10% of family members aged 25 or older without diabetes carried the variant (binomial p = 0.001). This contrasted with the other genes, where non-penetrance was less frequent (p > 0.06) (Table 2).

### Ultra-rare PTVs and damaging missense variants are enriched in *NEUROD1* and *PDX1* but not in *APPL1* and *WFS1*

**Figure 1:**
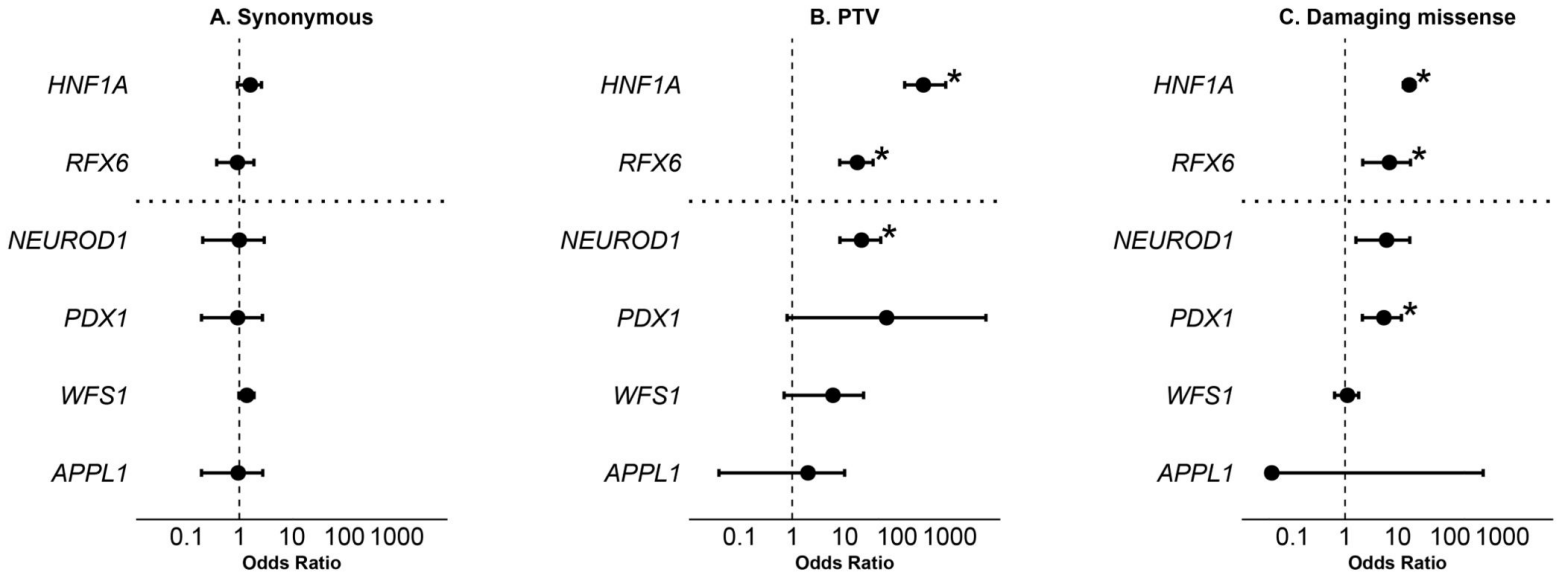
Gene-level burden analysis of ultra-rare variants in the MODY cohort The figure shows gene-level burden analyses comparing ultra-rare variants (MAF <1×10^−4^) between a European-ancestry MODY cohort (n=2,471) and UK Biobank controls (n=155,501). Analyses include: (A) synonymous variants (B) protein-truncating variants; and (C) damaging missense variants (REVEL >0.7). *HNF1A* and *RFX6* served as high- and low-penetrance positive controls, respectively. Asterisks (*) indicate significance after multiple testing corrections (p<0.002). We provide an odds ratio and a 95% confidence interval for each association.

Our frequency and co-segregation analysis examined specific, previously published variants and did not assess if other rare variants in these genes cause MODY. The enrichment of rare variants in a disease cohort provides strong genetic evidence of pathogenicity at the gene level. To do this, we used gene burden analyses to compare ultra-rare variants (MAF<1:10,000) in each gene between our MODY cases (n=2,471), and UK Biobank ancestry-matched controls (n=155,501). We found no enrichment of ultra-rare synonymous variants in any gene, confirming that our analysis is well calibrated, supporting our robust variant quality control process and also suggested that differences in sequencing technologies between cases and controls did not affect our results (Figure 1A, Supplementary Table 2). We observed significant enrichment of ultra-rare protein-truncating variants (PTVs) in *NEUROD1* in the MODY cohort (odds ratio [OR] - 21, 95% CI: 8-49, p = 3 × 10^−8^) (Figure 1B, Supplemental table 2). We did not see significant enrichment for PTVs in *PDX1, APPL1* or *WFS1* (all p > 0.03, Figure 1B, Supplemental Table 2). However, when we performed a sensitivity analysis that assessed NMD-escape PTVs (excluded from our initial analysis) we saw significant enrichment in *PDX1* (OR - 10, 95% CI: 4-24, p = 0.0001) but not in the other genes (all p > 0.9) (Supplemental figure 3). We observed enrichment for ultra-rare damaging missense variants for *NEUROD1* (OR - 6, 95% CI: 2-17, p = 0.005) and *PDX1* (OR - 5, 95% CI: 2-12, p = 0.0005) but no enrichment in *APPL1* or *WFS1* (p > 0.6). (Figure 1B, Supplemental Table 2) These findings support pathogenicity in *NEUROD1* and *PDX1*, but the odds ratios are closer to the low penetrance positive control *RFX6* than to the high penetrance control *HNF1A*.

### Multiple sensitivity analyses supported the main results that ultra-rare variants are enriched in *NEUROD1* and *PDX1* but not in *APPL1* and *WFS1*

We conducted multiple sensitivity analyses to support our primary results. We repeated our burden tests using an alternative control set from gnomAD v3.1.1 and observed consistent findings (Supplementary Table 3). To test whether our results depended on the exact frequency threshold, we repeated the burden analyses using a stricter allele frequency threshold (MAF<1:20,000), a more lenient threshold (MAF<1:5,000), and for PTVs, we also tested not using a frequency threshold. In all cases, we observed consistent results (Supplementary Tables 4 and 5, Supplementary figure 2) that *APPL1* and *WFS1* variants are not enriched in the MODY cohort, whereas *NEUROD1* and *PDX1* were at a level similar to *RFX6*. Finally, even when limiting to variants with MAC of 1, we did not observe enrichment for *APPL1* and *WFS1* (p>0.1).

### Domain-specific analysis increases *NEUROD1* and *PDX1* enrichment but not for *APPL1* and *WFS1*

**Figure 2:**
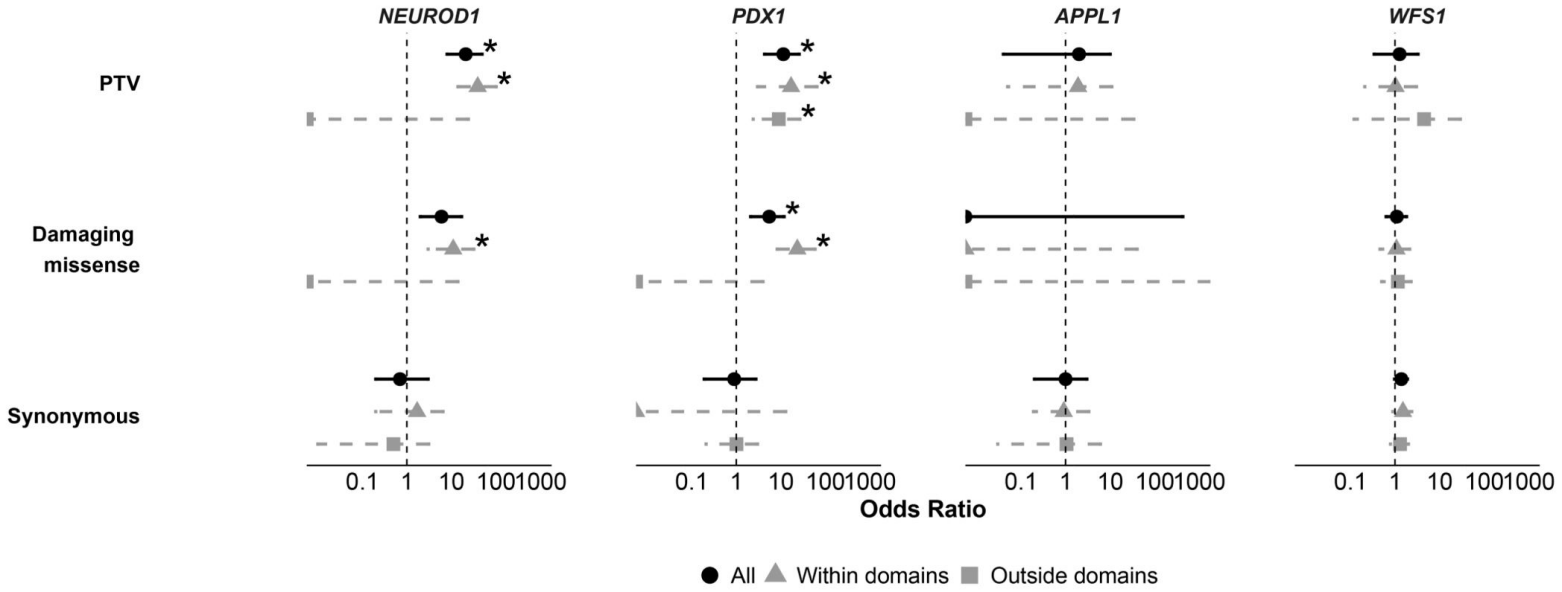
Functional domain-specific burden tests The figure shows gene-level burden analyses comparing ultra-rare variants (MAF <1×10^−4^) in a European-ancestry MODY cohort (n=2,471) against UK Biobank controls (n=155,501). We show data for A) protein-truncating variants, (B) damaging missense variants (REVEL >0.7), and (C) synonymous variants. We show data for all ultra-rare variants and split them by their location within or outside the functional domains. We provide an odds ratio and a 95% confidence interval for each association. Asterisks (*) indicate significance after multiple testing corrections (p<0.002).

We next assessed whether enrichment was restricted to specific functional regions of genes and whether including all variants, regardless of location, diluted the signal (domain information in Supplementary Table 6). We found that *WFS1* and *APPL1* showed no enrichment for PTVs or damaging missense variants whether they were within or outside functional domains (p > 0.05 for all comparisons, Figure 2). As an additional sensitivity analysis in *APPL1*, we specifically examined the phosphotyrosine binding domain that contains the originally reported p.L552* variant (29) and found no significant enrichment of ultra-rare PTVs in this region (p=0.4). In contrast, *NEUROD1* showed stronger enrichment for ultra-rare PTVs and ultra-rare damaging missense variants within functional domains (OR: 48, 11, p < 0.0008), while variants outside these domains showed no association (p=1) despite similar length of protein within and outside the domains (174 vs. 182 amino acids). Interesting, all ultra-rare damaging missense variants in *PDX1* in the MODY cohort were located within the functional domain, even though this region covered only 22% of the gene (Figure 2).

## Discussion

In our study using a large cohort of individuals with suspected MODY (n=2,471) and control cohorts (n=155,501), we show that ultra-rare heterozygous PTVs and damaging missense variants in *APPL1* and *WFS1* are not enriched within the MODY cohort, whereas *NEUROD1* and *PDX1* showed enrichment but at a level in line with our low-penetrance control gene *RFX6*.

The rare variants in *APPL1* are not associated with MODY. The enrichment of rare variants in a disease cohort provides strong evidence for gene-disease causality. Since the disease mechanism is expected to be loss of function, we expect to observe an enrichment of ultra-rare PTVs in the MODY cohort, similar to our positive controls, *HNF1A* and *RFX6*. However, we did not find any enrichment of ultra-rare PTVs in *APPL1*. This remains true even when focusing specifically on the functional domains, reinforcing the idea that PTVs in *APPL1* do not cause MODY. This is further supported by the prevalence of all PTVs in *APPL1* in gnomAD, which are too common to be a cause of MODY (combined allele count of 1 in 1,347 in gnomAD v4.1.0). We also did not observe an enrichment of damaging missense variants, which could potentially act through a dominant negative mechanism and might have produced a different result to PTVs. Additionally, in the *APPL1* pedigrees, 8 out of 23 individuals over the age of 25 carry the variant without having developed diabetes, five of whom are over the age of 35 (25). These data collectively suggest that the current genetic evidence does not support the causal role of *APPL1* in MODY. However, this does not imply that this gene is not biologically significant (25,31). It is also possible that rare variants may serve as a risk factor for type 2 diabetes, although the publicly available data on type 2 diabetes does not support this (P=0.60, https://t2d.hugeamp.org/gene.html?gene=APPL1).

Heterozygous variants in *WFS1* do not appear to be associated with MODY. This gene demonstrates complex genotype-phenotype relationships: recessive loss-of-function variants cause Wolfram syndrome and diabetes, C-terminal missense variants are associated with deafness, while common missense variants confer risk for type 2 diabetes. Notably, *de novo* missense variants can cause neonatal diabetes and congenital cataracts. The p.Trp314Arg variant, first reported in 2015 in a Finnish family with early-onset diabetes, was proposed to act through a loss-of-function or dominant-negative mechanism (10). Located in the first transmembrane domain (outside known functional regions), this variant was reported in a heterozygous state. Subsequent studies have described other heterozygous *WFS1* variants in diabetes cases, suggesting potential causality. However, our comprehensive analysis of a MODY cohort revealed no significant enrichment of ultra-rare PTVs or damaging missense variants in *WFS1*. This held true even when focusing on: (1) the functionally critical last exon, (2) known functional domains, or (3) variants with MAC=1 (comparable to p.Trp314Arg). We identified just one p.Trp314Arg carrier in our cohort (diagnosed at age 33, BMI 22.9, no family history of diabetes, no known other pathogenic variant). These findings indicate that not all ultra-rare damaging *WFS1* variants should be considered causative for MODY. The p.Trp314Arg remains a specific case and finding from this should not be generalised to all other variants. Further mechanistic studies are needed to understand why p.Trp314Arg is different from other variants in *WFS1*. Follow-up human studies in independent families are crucial to provide insight into this variant and its role in diabetes.

Evidence that variants in *NEUROD1* and *PDX1* have low-penetrance is present in the initial studies. Malecki *et al*. (27) reported all six individuals with the *NEUROD1* variant were diagnosed after 30 years of age, with the oldest in their 50s. Additionally, heterozygous parents of individuals with neonatal diabetes due to recessive *PDX1* variants do not consistently have diabetes (34). This is a similar pattern to *RFX6*, an established low-penetrance cause of MODY, which had adult individuals without diabetes within the reported pedigrees (26). Consistent with this pattern of low penetrance, our burden test results for *NEUROD1* and *PDX1* are more similar to those for *RFX6* than *HNF1A*. It is interesting that enrichment was seen only in the functional domains for *NEUROD1* and for *PDX1* missense variants highlighting their critical function. Another striking finding is that enrichment of PTVs in *PDX1* only occurs at the end of the gene within the NMD-escape region (which includes the functional domain). Our results are supported by previous functional work by Stoffers et al. (35), on p.Pro63Argfs*60, which creates a premature termination codon in the NMD-escape region and leads to expression of a truncated protein, suggesting it might cause disease by a dominant negative mechanism. These findings will need be replicated in further studies but based on our results, variants outside of the functional domain should be treated with caution.

Our study supports revising the pathogenicity classifications for these four genes. For *PDX1*, ClinGen currently assigns a genetic evidence score of 6.2/12 (based entirely on case-level data) plus 5 points for experimental evidence. Our gene burden analysis provides additional genetic evidence that would likely raise the total score above 12, warranting reclassification as “Definitive” for MODY. *NEUROD1* currently scores just 0.9 for genetic evidence and 3 for experimental evidence. Our findings could elevate its score above 7, supporting reclassification to “moderate.” While this justifies their inclusion on MODY testing panels, their low penetrance limits predictive value in unaffected individuals. *APPL1* is currently classified as “Limited” by ClinGen, while *WFS1* remains unclassified. Our gene burden results provide strong evidence against pathogenicity for MODY. We therefore recommend *APPL1* as “refuted” and restricting *WFS1* testing to specific missense variants with established evidence (10). These evidence-based revisions would optimize MODY testing strategies.

Our study has several limitations. First, our cohort was of European ancestry and we haven’t assessed other populations. While this is an important consideration, all initial reports of these four MODY-associated genes were derived from European populations, and no ancestry-specific effects have been documented for these genes to date, though future studies should explicitly evaluate these relationships in diverse ancestries. Second, although we utilized a large MODY cohort, our sample size had sufficient power only to detect associations with odds ratios ≥ 9.8 at an ultra-rare allele frequency of 0.0001. Consequently, we cannot exclude the possibility that *APPL1* or *WFS1* harbor extremely rare or low-penetrance MODY-causing variants. However, the relatively high population frequency of damaging variants in these genes suggests they are more likely to act as type 2 diabetes risk factors rather than monogenic causes of MODY.

In conclusion, we provide genetic evidence that *NEUROD1* and *PDX1* cause low penetrance MODY whereas variants in *APPL1* and *WFS1* do not cause MODY and should not be included in MODY genetic analysis. Our study highlights the importance of re-evaluating gene-disease relationships in light of available evidence, particularly the benefit of gene level rare variant analysis to support the genetic evidence of pathogenicity.

## Supplementary Material

Supplementary file

## Figures and Tables

**Table 1:** Allele frequencies of the first published variants in *NEUROD1, PDX1, APPL1* and *WFS1*

Gene	Variant	Allele count/total alleles (allele frequency) in gnomAD v2.1.1	Allele count in ancestry with maximum allele frequency in gnomAD v2.1.1 (ancestry)	Number and type of controls used in the original publication	References
*HNF1A*	p.P447L	3/249186 (1.2 x 10-5)	1/20812, 4.80 x 10^-5^ (Finnish)	55 (local controls)	(25)
	p.V380Sfs*4	0 (0)	0, 0		
	p.E548Rfs*112	0 (0)	0, 0		
	p.R131Q	1/251390 (3.98 x 10^-6^)	1/113698, 8.8 x 10^-6^ (non-Finnish European)		
	c.1768+1G>A	0 (0)	0, 0		
	c.1108-2A>G	0 (0)	0, 0		
*RFX6*	p.Q25*	1/31362 (3.19 x 10^-5^)	1/15422, 6.48 x 10^-5^ (non-Finnish European)	116,538 (33346 ExAC, 76152 gnomAD genomes, 7040 METSIM-Finnish)	(26)
	p.L292*	0 (0)	0, 0		
	p.H293Lfs	52/282332 (1.84 x 10^-4^)	51/25096, 2.03 x 10^-3^ (Finnish)		
	p.K351*	0 (0)	0, 0		
	p.R377*	1/250964 (3.98 x 10^-6^)	1/16252, 6.15 x 10^-5^ (African)		
	p.R652*	4/251320, 1.59 x 10^-5^)	1/16254, 6.15 x 10^-5^ (African)		
*NEUROD1*	p.H206Pfs*38	0 (0)^#^	0, 0^#^	96 (local controls)	(27)
*PDX1*	p.P63Rfs*60	2/138298 (1.45 x 10^-5^)	1/49632, 2.01 x 10^-5^ (non-Finnish European)	92 (local controls)	(28)
*WFS1*	p.W314R	0 (0)	0, 0	8,462 (1000 genomes project, 6464 from NHBLI exomes, 962 Finnish individuals from T2D-GENES consortium)	(10)
*APPL1*	p.L552*	0 (0)	0, 0	72,598 (61486 from ExAC, 6503 from Exome Sequencing Project, and an additional 4609 controls from PMID: 23633196)	(29)
	p.D94N	1/248002 (4.03 x 10^-6^)	1/112840, 8.86 x 10^-6^ (non-Finnish European)		

# variant filtered out in gnomAD

**Table 2:** Cosegregation in published pedigrees

Gene	No. of variants published	Variants (ref)	Combined LOD score	Proportion and number of family members with diabetes and variant	Binomial test P value (against expected proportion of 0.5)	Proportion and number of family members with variant but without diabetes by at least age 25	Binomial test P value (against expected proportion of 0.1)
*HNF1A*	7	p.G292Rfs*25, p.P447L, p.V380Sfs*4, p. R131Q, c.1768 + 1G>A, c.1108-2A>G (25)	9.6	1 (34/34)	1.64 x 10^-10^	0.17 (3/18)	0.4
*RFX6*	3	p.R181Q (26), p.S217F (21), p.L292* (26)	4.25	0.91 (20/22)	0.0001	0.23 (3/13)	0.1
*NEUROD1*	3	p.R103P (32), p.E110K (33),p.H206Pfs*38 (27)	8.1	1 (21/21)	9.57 x 10^-7^	0.27 (4/15)	0.06
*PDX1*	1	p.P63Rfs*60 (28)	3.43	0.89 (8/9)	0.04	0.18 (2/9)	0.2
*WFS1*	1	p.W314R (10)	2.1	1 (7/7)	0.02	0(0/4)	1
*APPL1*	2	p.L552*, p.D94N (29)	3.3	0.92 (11/12)	0.006	0.35 (8/23)	0.001

## Article highlights

- **Why did we undertake this study?**
  Testing genes with limited evidence of causality risks misdiagnosis of MODY.
- **What is the specific question(s) we wanted to answer?**
  Do rare variants in *PDX1, NEUROD1, APPL1*, and *WFS1* cause MODY?
- **What did we find?**
  Rare damaging variants in *APPL1* and *WFS1* are not enriched in a MODY cohort, but those in *PDX1* and *NEUROD1* are, at a level similar to low-penetrance MODY genes.
- **What are the implications of our findings?**
  *PDX1* and *NEUROD1* should be included in MODY gene panels, while heterozygous *APPL1* and *WFS1* variants should not be reported as causes of MODY.
