# Supplementary material for: Rare variants in NEUROD1 and PDX1 are low penetrance causes of MODY, whereas those in APPL1 and WFS1 are not associated with MODY
Source: Diabetes. Author manuscript; Available in PMC 2025 Nov 1. (PMC7617998; doi:10.2337/db25-0442)
Supplement: Supplementary file [file EMS207696-supplement-Supplementary_file.docx]

**Supplemental Results**

Supplemental table 1: Characteristics of MODY cohort

Median (IQR) for continuous variable and n (%) for categorical data

| **Characteristics** | **MODY cohort** |
| --- | --- |
| N | 2471 |
| Age of diagnosis of diabetes, years | 23 (16-31) |
| Female Sex | 59.4% |
| Age at recruitment, years | 32 (22-42) |
| BMI, kg/m^2^ | 26 (22.7-30.2) |
| Parents with diabetes | 67.5% |
| HbA1c, % | 7.8 (6.7-10) |
| On Insulin treatment | 47.6% |
| European ancestry (genetically determined) | 100% |

**Supplemental table 2: Gene burden test for variants with MAF < 0.0001 with UK biobank as controls**

| **Variant type** | **Gene** | **Allele count  in MODY  cohort** | **Allele count  in population  cohort  (UK biobank)** | **Odds ratio (95% CI)** | **P Value** |
| --- | --- | --- | --- | --- | --- |
| Rare PTVs | *HNF1A* | 35 | 7 | 317 (139-845) | 1.36 x 10^-56^ |
| (MAF < 0.0001) | *RFX6* | 11 | 38 | 17 (8-35) | 3.55 x 10^-10^ |
|  | *NEUROD1* | 8 | 23 | 21 (8-49) | 2.80 x 10^-8^ |
|  | *PDX1* | 1 | 1 | 63 (0.8-4941) | 0.03 |
|  | *WFS1* | 2 | 22 | 6 (0.7-23) | 0.05 |
|  | *APPL1* | 1 | 46 | 2 (0.04-10) | 0.4 |
| Rare damaging missense variants | *HNF1A* | 78 | 281 | 17 (13-22) | 1.14 x 10^-61^ |
| (MAF < 0.0001) | *RFX6* | 5 | 43 | 7 (2-18) | 0.001 |
|  | *NEUROD1* | 4 | 39 | 6 (2-17) | 0.005 |
|  | *PDX1* | 7 | 77 | 5 (2-12) | 0.0005 |
|  | *WFS1* | 16 | 868 | 1 (0.6-2) | 0.6 |
|  | *APPL1* | 0 | 2 | 0 (0-429) | 1 |
| All PTVs | *HNF1A* | 35 | 7 | 317 (139-845) | 1.36 x 10^-56^ |
|  | *RFX6* | 11 | 38 | 17 (8-35) | 3.55 x 10^-10^ |
|  | *NEUROD1* | 8 | 23 | 21 (8-49) | 2.80 x 10^-8^ |
|  | *PDX1* | 1 | 1 | 63 (0.8-4941) | 0.03 |
|  | *WFS1* | 16 | 844 | 1 (0.7-2) | 0.5 |
|  | *APPL1* | 6 | 254 | 2 (0.7-4) | 0.1 |
| Rare synonymous variants | *HNF1A* | 16 | 598 | 2 (1-3) | 0.08 |
| (MAF < 0.0001) | *RFX6* | 7 | 462 | 1 (0.4-2) | 1.00 |
|  | *NEUROD1* | 3 | 181 | 1 (0.2-3) | 1.00 |
|  | *PDX1* | 3 | 195 | 1 (0.2-3) | 1.00 |
|  | *WFS1* | 39 | 1695 | 1 (0.98-2) | 0.05 |
|  | *APPL1* | 3 | 252 | 1 (0.2-3) | 1.00 |

**Supplemental table 3: Gene burden test for variants with MAF < 0.0001 with gnomAD v3 as controls**

| **Variant type** | **Gene** | **Allele count  in MODY  cohort** | **Allele count  in population  cohort  (gnomAD)** | **Odds ratio (95% CI)** | **P Value** |
| --- | --- | --- | --- | --- | --- |
| Rare PTVs | *HNF1A* | 36 | 3 | 166 (53-845) | 5.27 x 10^-39^ |
| (MAF < 0.0001) | *RFX6* | 9 | 9 | 13 (5-38) | 1.14 x 10^-6^ |
|  | *NEUROD1* | 8 | 6 | 18 (5-62) | 1.22x10^-6^ |
|  | *PDX1* | 1 | 0 | Inf (0-Inf) | 0.07 |
|  | *WFS1* | 2 | 6 | 5(0.5-26) | 0.1 |
|  | *APPL1* | 1 | 5 | 3 (0.1-31) | 0.3 |
| Rare damaging missense variants | *HNF1A* | 78 | 61 | 17 (12-24) | 3.99 x 10^-52^ |
| (MAF < 0.0001) | *RFX6* | 5 | 1 | 66 (7-3067) | 0.00001 |
|  | *NEUROD1* | 4 | 9 | 6 (1-21) | 0.01 |
|  | *PDX1* | 7 | 9 | 10 (3-31) | 0.0001 |
|  | *WFS1* | 19 | 182 | 1 (1-2) | 0.2 |
|  | *APPL1* | 0 | 0 | 0 (0-Inf) | 1 |
| All PTVs | *HNF1A* | 36 | 3 | 166 (53-845) | 5.27E-39 |
|  | *RFX6* | 9 | 9 | 13 (5-38) | 1.14 x 10^-6^ |
|  | *NEUROD1* | 8 | 6 | 18 (5-62) | 1.22 x 10^-6^ |
|  | *PDX1* | 1 | 0 | Inf (0-Inf) | 0.07 |
|  | *WFS1* | 3 | 16 | 3 (0.5-9) | 0.1 |
|  | *APPL1* | 6 | 54 | 2 (0.7-5) | 0.1 |
| Rare synonymous variants | *HNF1A* | 15 | 132 | 2 (1-3) | 0.14 |
| (MAF < 0.0001) | *RFX6* | 9 | 102 | 1 (1-2) | 0.58 |
|  | *NEUROD1* | 3 | 59 | 1 (0.1-2) | 0.80 |
|  | *PDX1* | 3 | 49 | 1 (0.2-3) | 1.00 |
|  | *WFS1* | 35 | 348 | 1 (1-2) | 0.13 |
|  | *APPL1* | 2 | 56 | 1 (0.1-2) | 0.77 |

**Supplemental table 4: Sensitivity analysis burden test (MAF<0.00005) in MODY cohort (n = 2,471) and UK Biobank (n = 155,501)**

| **Variant type** | **Gene** | **Allele count  in MODY  cohort** | **Allele count  in population  cohort  (UK biobank)** | **Odds ratio (95% CI)** | **P Value** |
| --- | --- | --- | --- | --- | --- |
| Rare PTVs | *HNF1A* | 35 | 7 | 317 (139-845) | 1.36 x 10^-56^ |
| (MAF < 0.00005) | *RFX6* | 11 | 38 | 17 (8-35) | 3.55 x 10^-10^ |
|  | *NEUROD1* | 8 | 23 | 21 (8-49) | 2.80 x 10^-8^ |
|  | *PDX1* | 1 | 1 | 63 (0.8-4941) | 0.03 |
|  | *WFS1* | 2 | 22 | 6 (0.7-23) | 0.05 |
|  | *APPL1* | 1 | 46 | 2 (0.04-10) | 0.4 |
| Rare damaging missense variants | *HNF1A* | 77 | 240 | 19 (15-25) | 7.24 x 10^-65^ |
| (MAF < 0.00005) | *RFX6* | 5 | 43 | 7 (2-18) | 0.001 |
|  | *NEUROD1* | 4 | 39 | 6 (2-17) | 0.005 |
|  | *PDX1* | 7 | 32 | 13 (5-31) | 2.96 x 10^-6^ |
|  | *WFS1* | 12 | 533 | 1 (1-2) | 12 |
|  | *APPL1* | 0 | 2 | 0 (0-429) | 1 |
| Rare synonymous variants | *HNF1A* | 11 | 395 | 2 (1-3) | 0.11 |
| (MAF < 0.00005) | *RFX6* | 5 | 268 | 1 (0.4-3) | 0.64 |
|  | *NEUROD1* | 2 | 122 | 1 (0.1-4) | 1.00 |
|  | *PDX1* | 3 | 178 | 1 (0.2-3) | 0.77 |
|  | *WFS1* | 27 | 964 | 2 (1-2) | 0.01 |
|  | *APPL1* | 1 | 209 | 0 (0-2) | 0.53 |

**Supplemental table 5: Sensitivity analysis burden test (MAF<0.0002) in MODY cohort (n = 2,471) and UK Biobank (n = 155,501)**

| **Variant type** | **Gene** | **Allele count  in MODY  cohort** | **Allele count  in population  cohort  (UK biobank)** | **Odds ratio (95% CI)** | **P Value** |
| --- | --- | --- | --- | --- | --- |
| Rare PTVs | *HNF1A* | 35 | 7 | 317 (139-845) | 1.36 x 10^-56^ |
| (MAF < 0.0002) | *RFX6* | 11 | 38 | 17 (8-35) | 3.55 x 10^-10^ |
|  | *NEUROD1* | 8 | 23 | 21 (8-49) | 2.80 x 10^-8^ |
|  | *PDX1* | 1 | 1 | 63 (0.8-4941) | 0.03 |
|  | *WFS1* | 2 | 22 | 6 (0.7-23) | 0.05 |
|  | *APPL1* | 1 | 46 | 2 (0.04-10) | 0.4 |
| Rare damaging missense variants | *HNF1A* | 81 | 372 | 13 (10-17) | 6.56 x 10^-57^ |
| (MAF < 0.0002) | *RFX6* | 5 | 43 | 7 (2-18) | 0.001 |
|  | *NEUROD1* | 4 | 39 | 6 (2-17) | 0.005 |
|  | *PDX1* | 7 | 77 | 6 (2-12) | 0.0005 |
|  | *WFS1* | 34 | 1296 | 2 (1-2) | 0.01 |
|  | *APPL1* | 0 | 2 | 0 (0-429) | 1 |
| Rare synonymous variants | *HNF1A* | 22 | 882 | 2 (1-2) | 0.06 |
| (MAF < 0.0002) | *RFX6* | 11 | 678 | 1 (0.5-2) | 1.00 |
|  | *NEUROD1* | 4 | 218 | 1 (0.3-3) | 0.79 |
|  | *PDX1* | 3 | 240 | 1 (0.2-2) | 1.00 |
|  | *WFS1* | 46 | 2233 | 1 (1-2) | 0.16 |
|  | *APPL1* | 4 | 393 | 1 (0.2-2) | 1.00 |

**Supplemental figure 1: Gene burden test for all protein truncating variants in MODY cohort (n = 2,471) and UK Biobank (n = 155,501).** Analysis includes all protein-truncating variants, with no allele frequency threshold. *HNF1A* and *RFX6* served as high- and low-penetrance positive controls, respectively. Asterisks (*) indicate significance after multiple testing corrections (p<0.002). We provide an odds ratio and a 95% confidence interval for each association.

**Supplemental table 6: Functional domains used in domain specific burden test analyses**

| **Gene** | **Uniprot ID** | **Domains: Interpro* representative domains + Pfam domain** | **Domain sizes combined/gene size** |
| --- | --- | --- | --- |
| *APPL1* | Q9UKG1 | BAR domain of APPL family: 7-247, Adaptor protein containing PH domain: 252-376, Phosphotyrosine-binding domain: 497-636 | 503/709 (71%) |
| *WFS1* | O76024 | Wolframin Sel1-like repeat: 99-133, 137-175, Wolframin EF-hand domain: 177-255, Wolframin cysteine-rich domain: 666-769, Wolframin C-terminal OB-fold domain: 770-889 | 372/890 (42%) |
| *NEUROD1* | Q13562 | Basic helix-loop-helix: 75-160, Neuronal helix-loop-helix transcription factor: 160-284 | 209/356 (59%) |
| *PDX1* | P52945 | Homeodomain: 146-208 | 62/283 (22%) |

*InterPro 104.0. Accessed on 5/3/2025

**
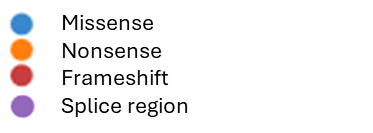
**

***NEUROD1***

**
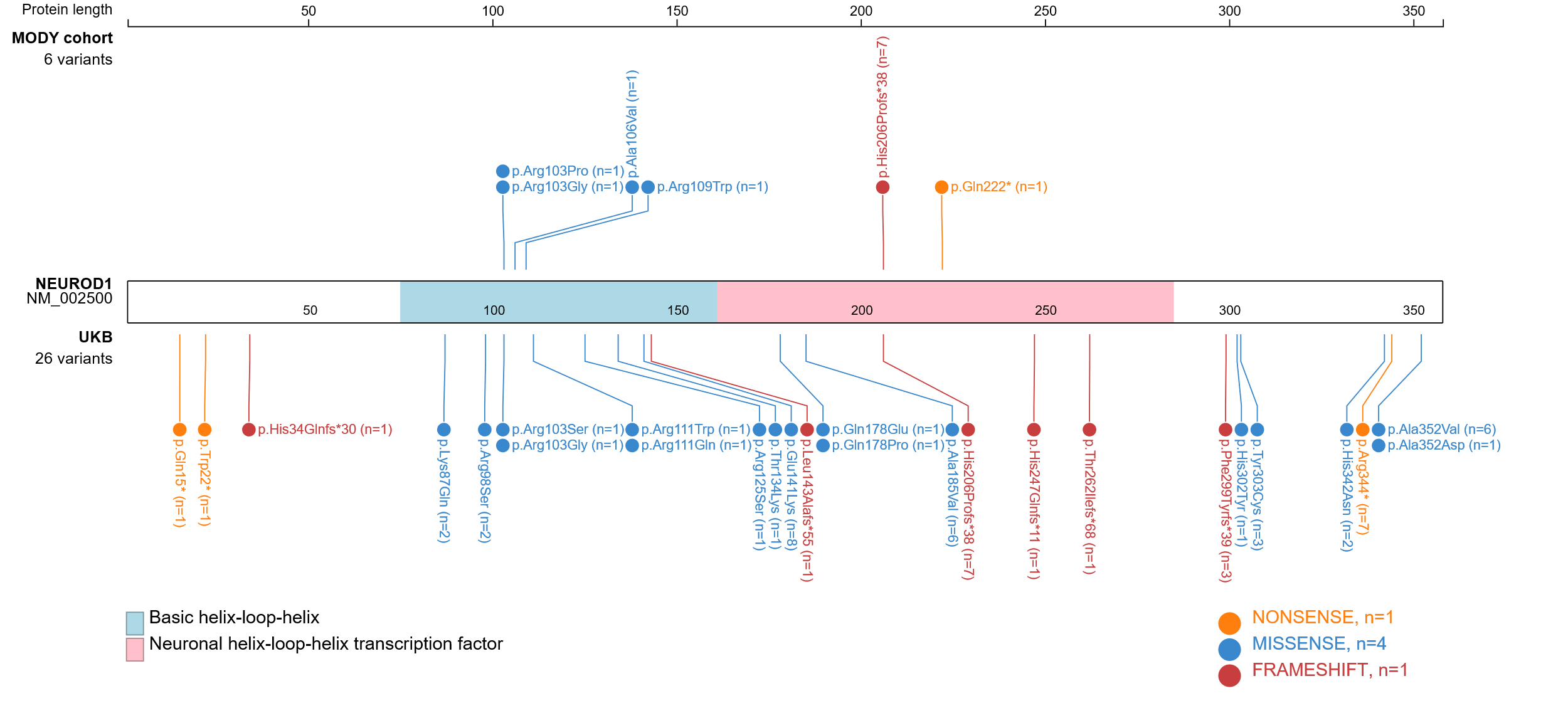
**

**
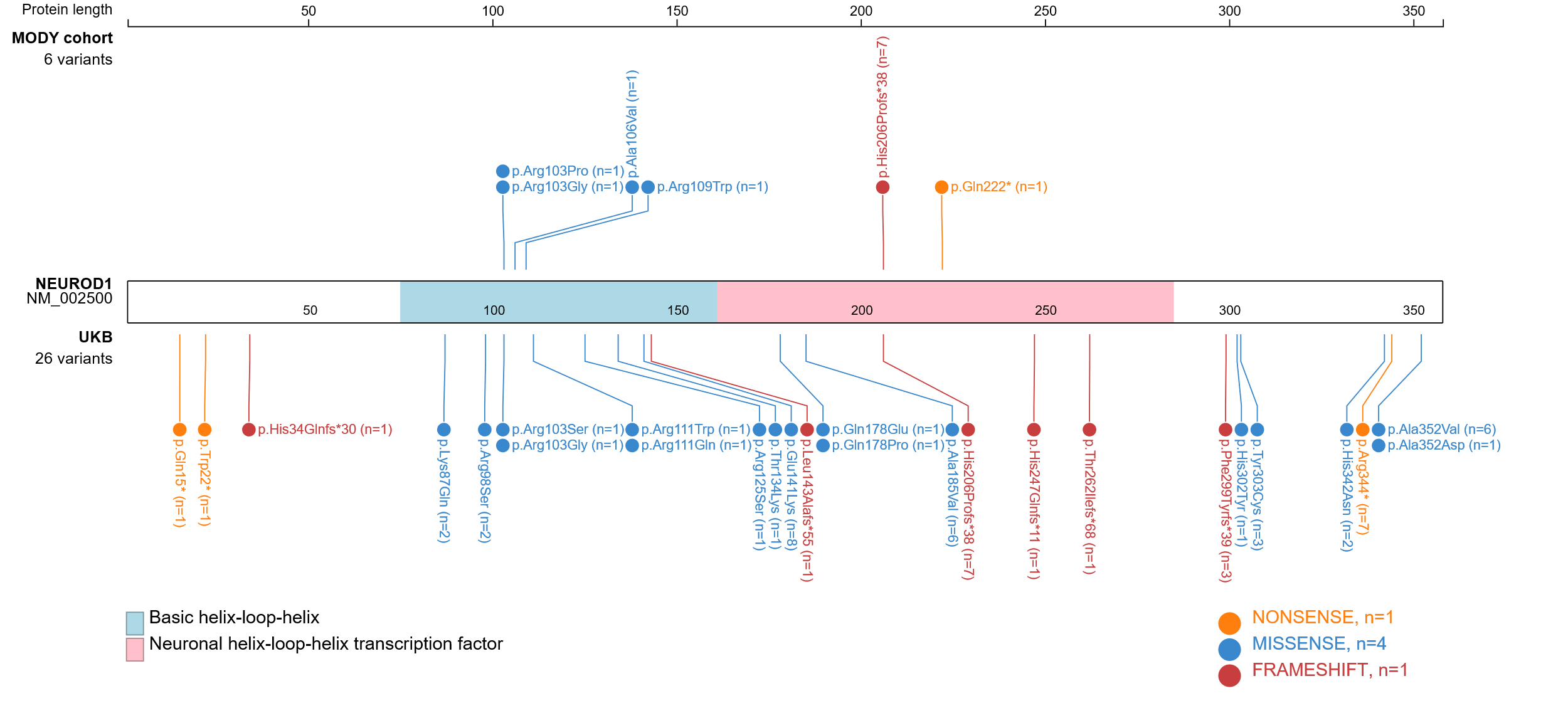
**

***PDX1***


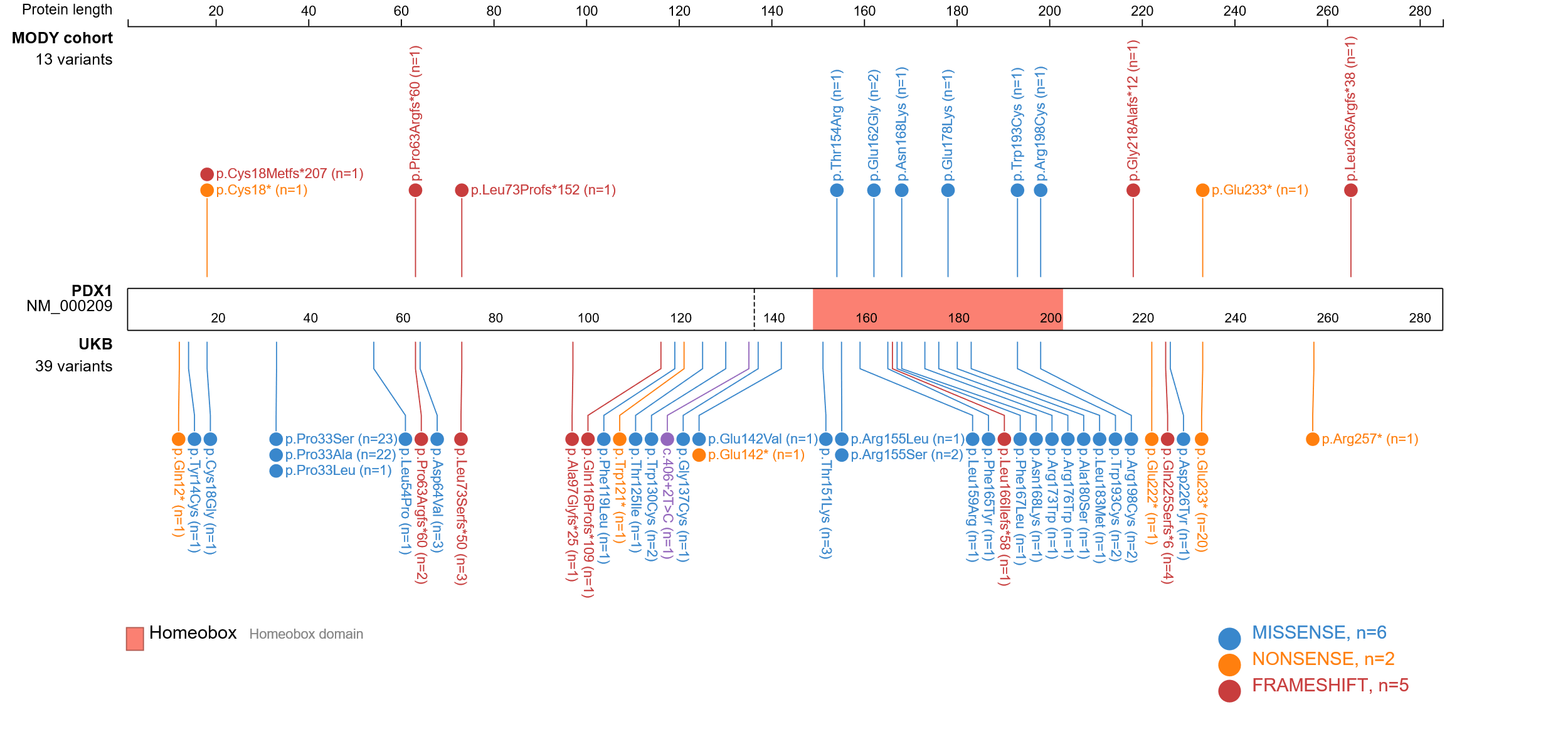


**
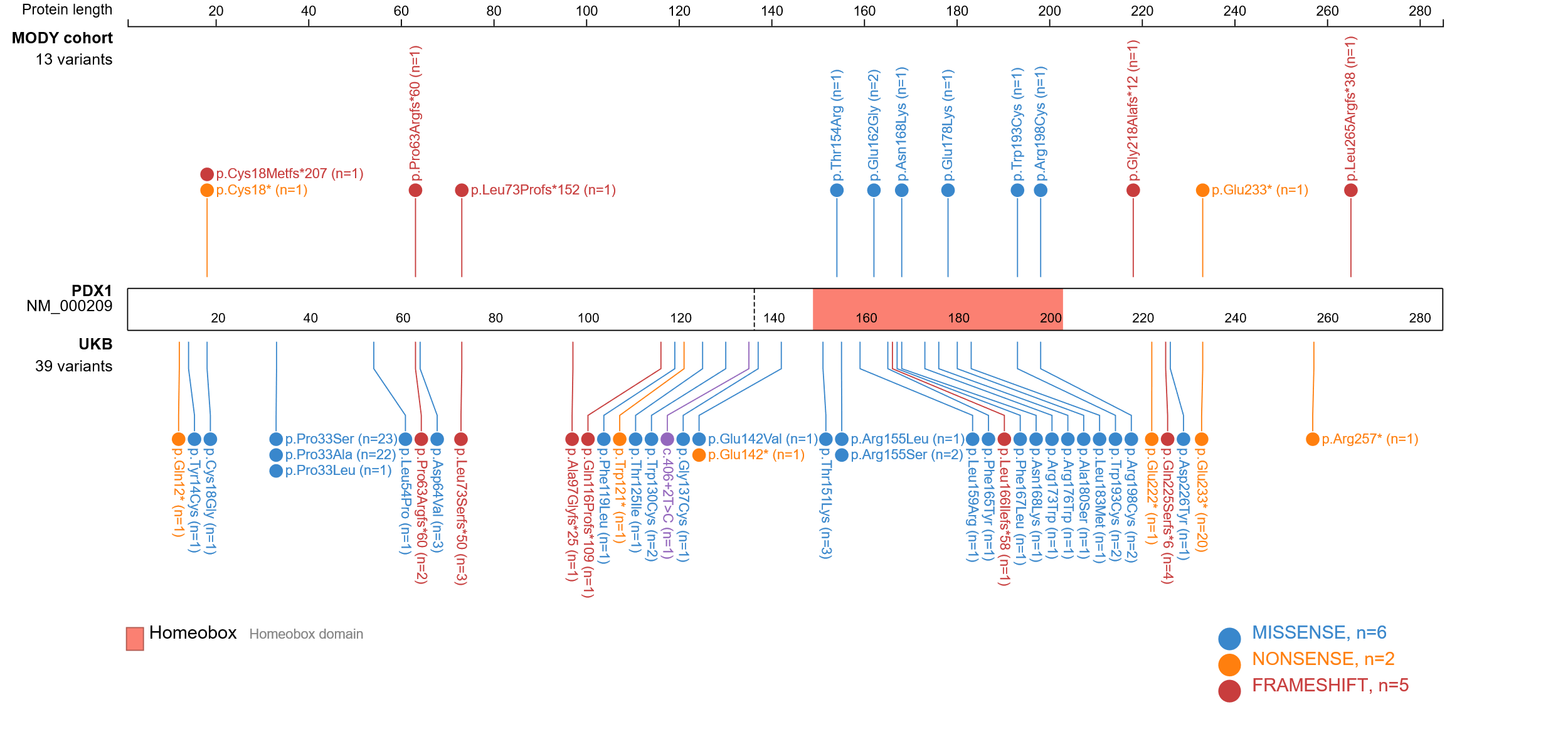
**

***WFS1***


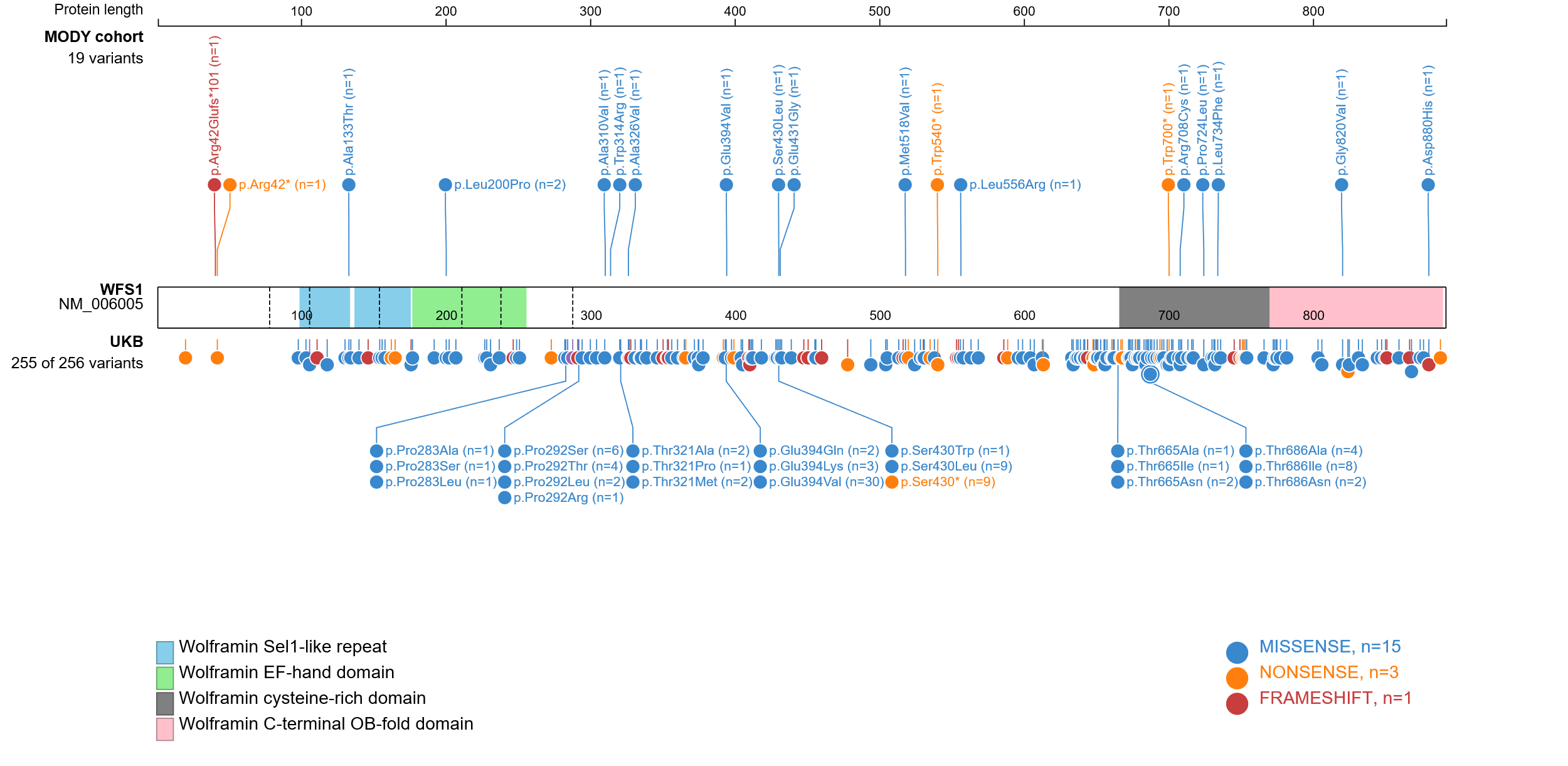


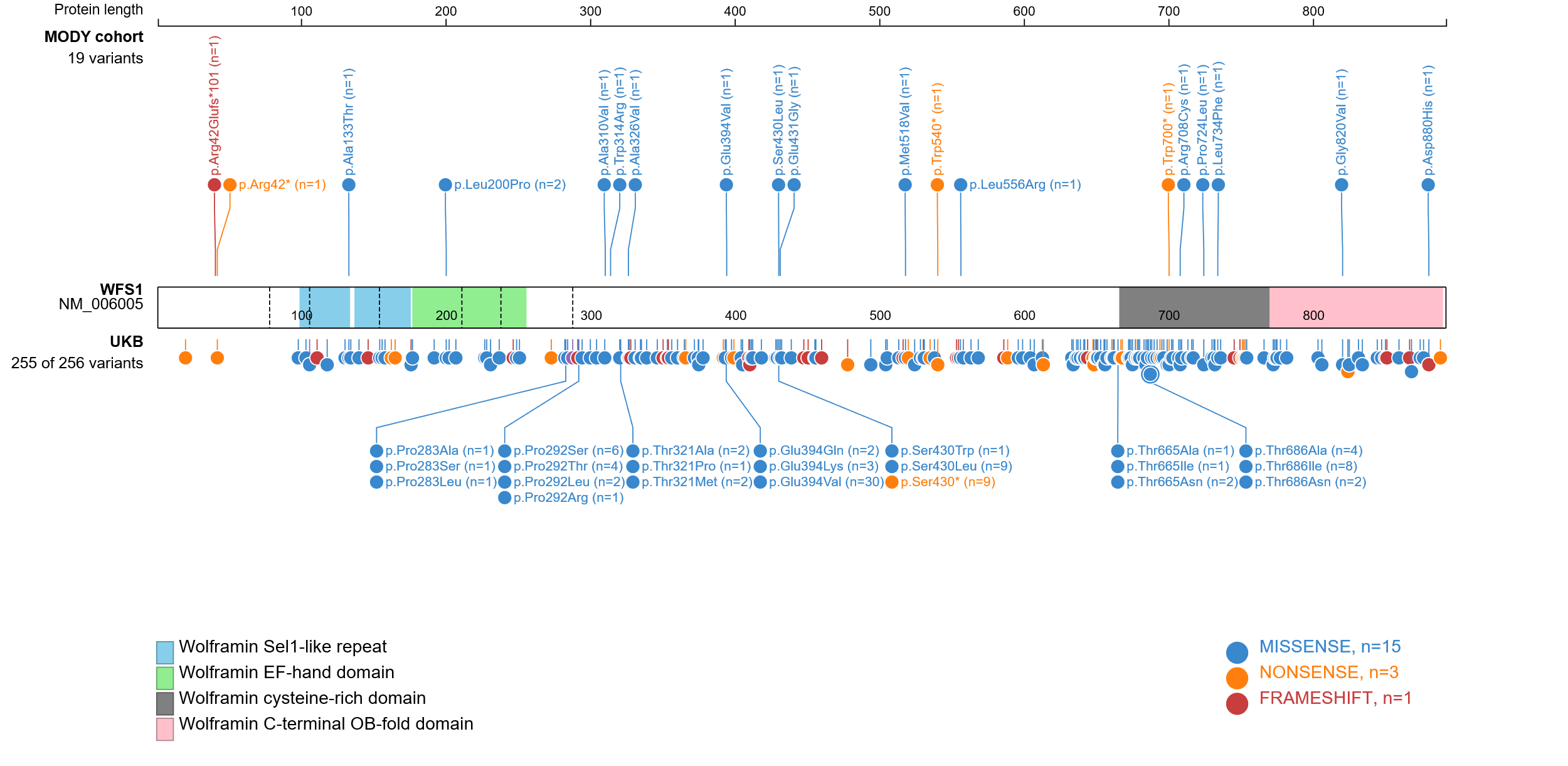


***APPL1***

**
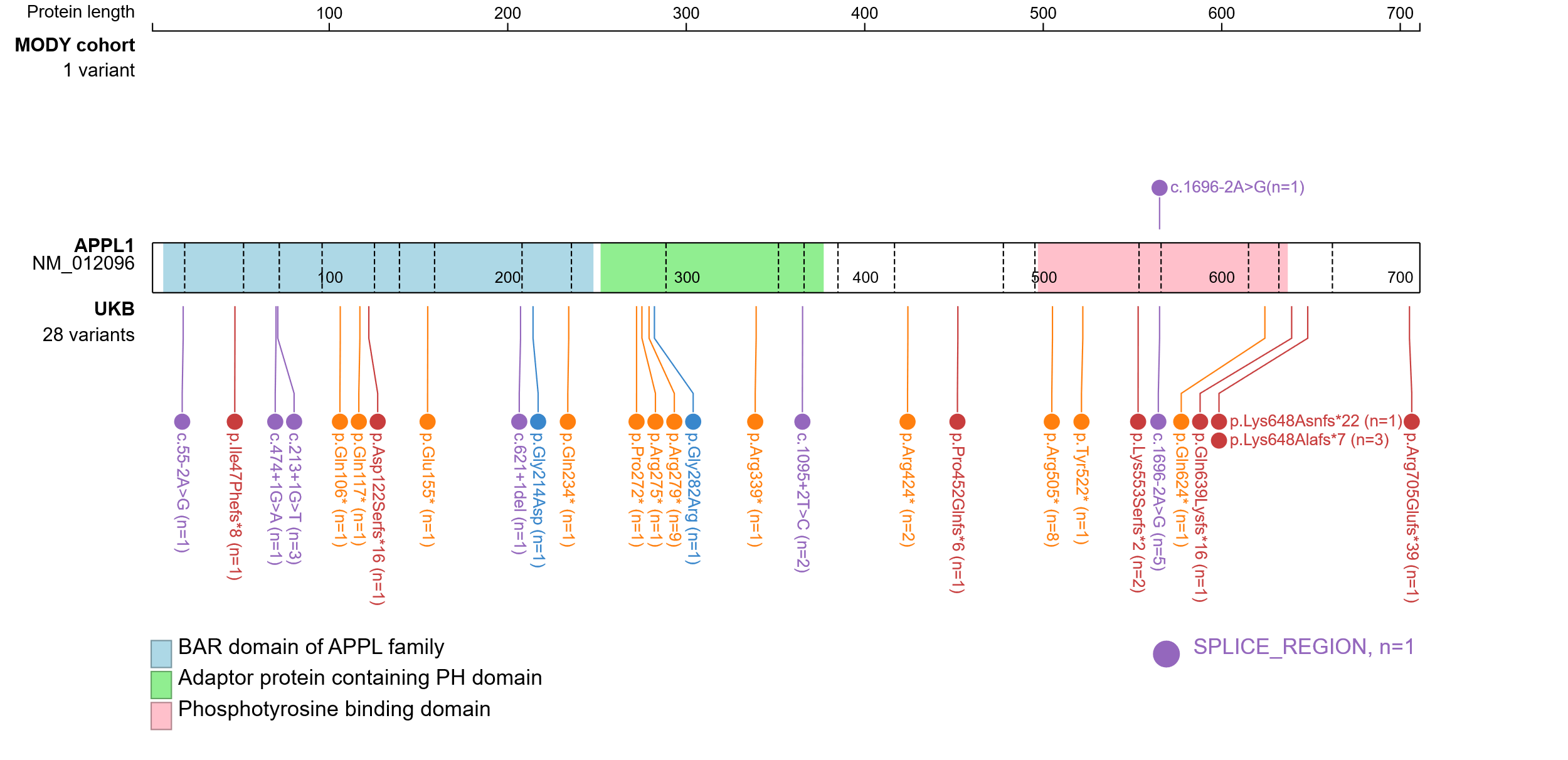
**


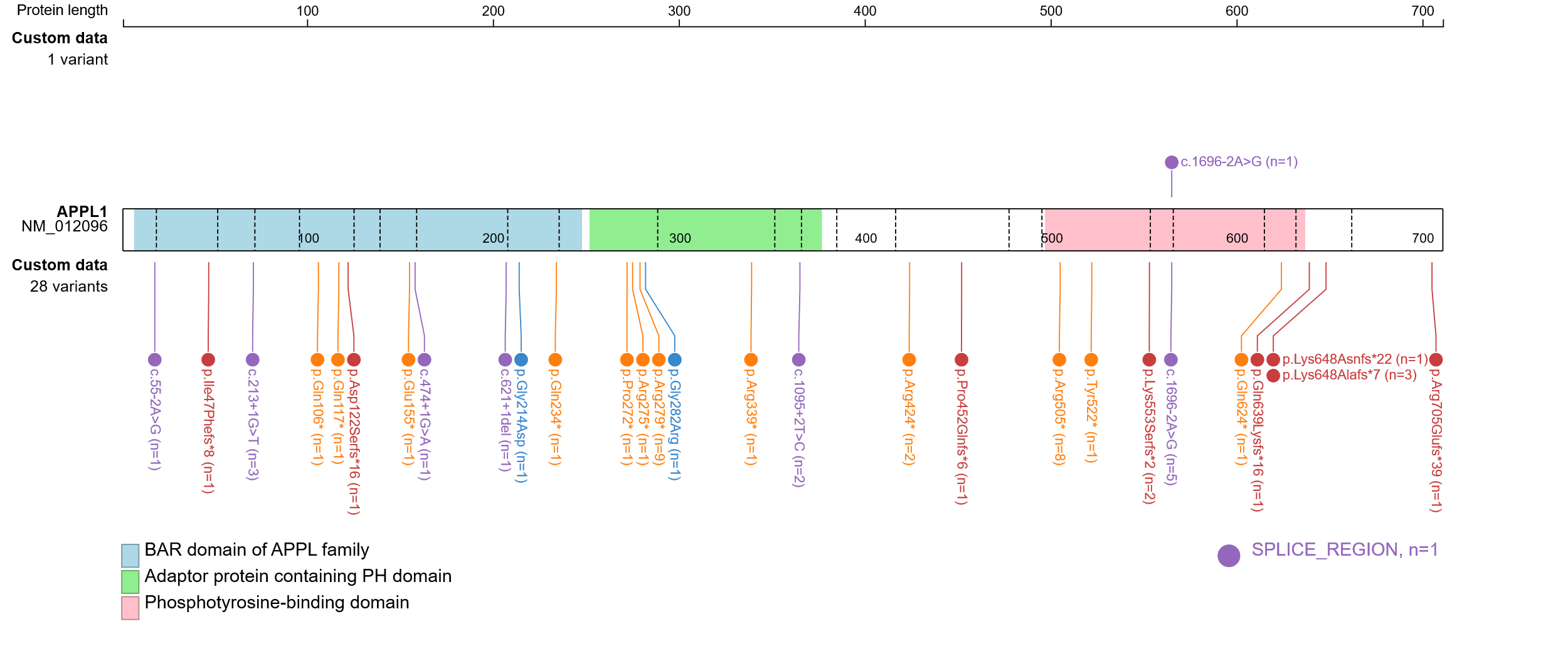


**Supplementary figure 2: Distribution of rare variants (MAF < 0.0001) in *NEUROD1*, *PDX1*, *APPL1* and *WFS1*.** This includes protein truncating variants split into nonsense, frameshift and splice region variants, and missense variants with a REVEL score > 0.7. The functional domains within each gene have been indicated. Variants identified in the MODY cohort are shown above the gene, while those from the UK Biobank are displayed below. Figures were generated using ProteinPaint (<https://proteinpaint.stjude.org/>).

**Supplementary figure 3: Gene burden tests for rare (MAF < 0.0001) protein truncating variants (PTV) and synonymous variants within and outside the NMD regions in *PDX1*, *APPL1* and *WFS1*.** This compares the MODY cohort (n = 2,471) and UK Biobank (n = 155,501). NMD refers to nonsense-mediated decay. The NMD-escape region analysis included PTVs in the last exon and the last 50bp of the penultimate exon of our genes of interest, while the NMD region analysis included PTVs in the rest of the gene. Synonymous variants were tested as a sensitivity analysis. *NEUROD1* was excluded because it is a single exon gene with all positions predicted to escape NMD.
